# Development of an RT-qPCR and a Next-Generation Sequencing approach to assess viral shedding of NDV-based SARS-CoV-2 variant vaccines

**DOI:** 10.1128/spectrum.00653-25

**Published:** 2025-11-25

**Authors:** Marta Boza, Adam Abdeljawad, Stefan Slamanig, Nicholas Lemus, Tsoi Ying Lai, William Shea, Weina Sun, Peter Palese, Irene González-Domínguez

**Affiliations:** 1 Department of Microbiology, Icahn School of Medicine at Mount Sinai 5925, New York, New York, USA; 2 Swammerdam Institute for Life Sciences, University of Amsterdam 1234, Amsterdam, the Netherlands; 3 Department of Medicine, Icahn School of Medicine at Mount Sinai 5925, New York, New York, USA; Institute of Microbiology Chinese Academy of Sciences, Beijing, China

**Keywords:** viral vector vaccine, live-attenuated vaccine, mucosal immunity

## Abstract

Live-attenuated intranasal vaccines offer great promise to generate mucosal immunity and reduce breakthrough infections after vaccination. Previously, we have developed a Newcastle disease virus (NDV)-based vaccine expressing the spike (S) protein of severe acute respiratory syndrome coronavirus 2 (SARS-CoV-2), named NDV-HXP-S. This vaccine can be administered intranasally as a live-attenuated vector to provide both systemic and mucosal immunity. With the appearance of new SARS-CoV-2 variants of concern, new NDV-HXP-S variant-based vaccines have been developed. These new NDV-HXP-S variant vaccines can be combined in a multivalent formulation to extend their protection against other SARS-CoV-2 variants of concern. One of the main concerns when administering a live-attenuated vector intranasally is the possible vaccine shedding after vaccination. In the present work, we have developed a reverse transcriptase-quantitative polymerase chain reaction (RT-qPCR) method to evaluate NDV-HXP-S *in vitro* and *in vivo* along with an amplicon-based next-generation sequencing technique to quantify the relative percentage of each variant vaccine in the multivalent formulation. Both techniques were validated following the recommendations of the Food and Drug Administration and the European Medicines Agency. Finally, we applied both methods in *in vitro* cell culture experiments. The quantification of the NDV-based vaccines by RT-qPCR and the relative percentage of each variant vaccine were confirmed using a prototype trivalent formulation expressing the Ancestral (Wuhan), Beta, and Delta SARS-CoV-2 spikes. The present results will help the study of virus shedding in future clinical trials.

**IMPORTANCE:**

Live-attenuated intranasal vaccines offer great promise to generate mucosal immunity and reduce breakthrough infections after vaccination. In this work, we have developed a combined reverse transcriptase-quantitative polymerase chain reaction and next-generation sequencing-directed approach to assess the viral shedding of multivalent Newcastle disease virus-based severe acute respiratory syndrome coronavirus 2 vaccines. This new formulation will be evaluated as a mucosal booster in phase I clinical trials next year.

## INTRODUCTION

**Fig 1:**
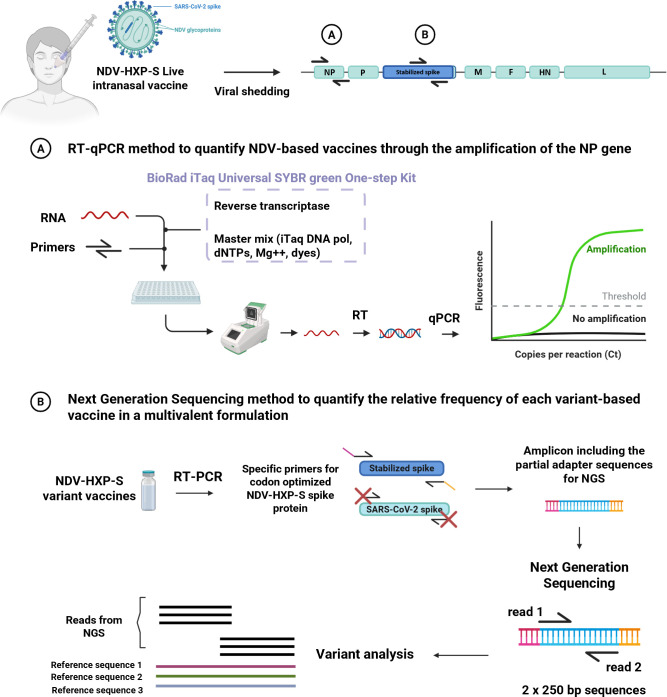
Validation of a reverse transcriptase-quantitative polymerase chain reaction (RT-qPCR) and a receptor-binding domain-based next-generation sequencing (NGS) method to quantify NDV-HXP-S vaccines. NDV-HXP-S vaccine can work as a mucosal vaccine when administered intranasally. The NDV genome is composed of six genes (nucleoprotein [NP], phosphoprotein [P], matrix protein [M], fusion protein [F], hemagglutinin-neuraminidase protein [HN], and large protein [L]). NDV-HXP-S vaccine contains the codon-optimized spike protein of SARS-CoV-2 variant vaccines. Two different methods were validated to study the viral shedding and vaccine composition of NDV-HXP-S variant vaccines in *in vitro* and *in vivo*. (**A**) A one-step RT-qPCR method to quantify NDV-based vaccines through the amplification of the NP gene. (**B**) An NGS method to quantify the relative frequency of each variant-based vaccine in a multivalent formulation. Figure created with Biorender.

Severe acute respiratory syndrome coronavirus 2 (SARS-CoV-2) is the causative agent of the coronavirus disease 2019 (COVID-19) (1, 2). In previous work, our groups have developed a live-attenuated viral vector vaccine expressing the spike (S) protein of SARS-CoV-2 based on the Newcastle disease virus (NDV) named NDV-HXP-S (3). This vaccine contains the mammalian codon-optimized sequence of the S protein inserted between the phosphoprotein (P) and the matrix (M) genes of the NDV (Fig. 1). Compared to other vaccine platforms, NDV-HXP-S presents several advantages: (i) it is based on the lentogenic LaSota strain, an avirulent and non-human pathogen that has a long safety record as veterinary vaccine and human oncolytic vector (4); (ii) NDV can be produced at high yields at a very low cost in embryonated chicken eggs; (iii) it can be administered as live viral vector vaccine via the intranasal route, conferring both humoral and mucosal immunity; and (iv) its high immunogenicity has been demonstrated in several preclinical and clinical studies (3, 5–7). With the appearance of SARS-CoV-2 variants of concern (VOC) which threaten the protection conferred by the prototype vaccines, new NDV-HXP-S variant-based vaccines have been developed. To date, NDV-HXP-S vaccines expressing the spike protein of Ancestral (Wuhan), Beta, Gamma, Delta, Omicron BA.1, Omicron BA.5, BQ.1.1, and XBB.1.5 have been produced (8–10). Furthermore, our groups have optimized a multivalent vaccine, combining these vectors in a single formulation, which demonstrated extended protection against mismatched SARS-CoV-2 VOCs, not present in the formulation (8).

Given the great potential of this new formulation as a mucosal multivalent vaccine, we have developed two methods to (i) quantitatively evaluate NDV-HXP-S *in vitro* and *in vivo* and (ii) to quantify the different relative percentages of each NDV-HXP-S vaccine in a multivalent formulation. Regulatory guidelines developed by the Food and Drug Administration (FDA) and the European Medicines Agency recommend nucleic acid-based assays to analyze vector biodistribution, vector shedding, and vector-derived gene expression due to their superior sensitivity and specificity at a wide dynamic range (11). Following these recommendations, we developed a one-step reverse transcriptase-quantitative polymerase chain reaction (RT-qPCR) method to quantify a small region of the nucleoprotein (NP) gene of the NDV (Fig. 1A). International Council for Harmonization (ICH) guideline M10 (12) and the FDA recommendations (13) were followed to validate the RT-qPCR method. The identity, linearity, sensitivity, specificity, precision, and reproducibility were optimized to meet the acceptance criteria. To differentiate each NDV-HXP-S variant vaccine, we developed an amplicon-based next-generation sequencing (NGS) method (Fig. 1B) (14). We selected the receptor-binding domain (RBD) region of the spike protein, which accumulates the highest number of mutations among VOCs, and performed NGS to quantify the frequency of each variant in a multivalent formulation. The identity, sensitivity, precision, and reproducibility were studied following the acceptance criteria. Finally, both techniques were applied in *in vitro* cell culture models.

## MATERIALS AND METHODS

### NDV-HXP-S production in embryonated chicken eggs

Ten-day-old specific pathogen-free (SPF) embryonated chicken eggs (AVSBio, Norwich, CT, USA) were infected with vaccine seed viruses to produce NDV-HXP-S variant vaccines. The incubation time and plaque-forming units per egg were optimized for each NDV-HXP-S variant. One hundred microliters of each master virus seed containing 10–300 plaque-forming units per egg was injected into the allantoic cavity of each egg. Eggs were incubated at 37°C, according to their incubation time and cooled at 4°C overnight. The specific growth conditions are reported in Table S1. Allantoic fluid (AF) was harvested from cooled eggs and subsequently clarified by centrifugation at 2,000 × *g* at 4°C for 30 minutes. NDV-HXP-S production was confirmed by hemagglutination (HA) assays. The clarified AF was aliquoted and stored at −80°C. Live viruses harvested in AF were pelleted through a 20% sucrose cushion in PBS (pH = 7.4) by ultracentrifugation in a Beckman L7-65 ultracentrifuge at 25,000 rpm, which corresponds to 107,000 × *g* or 113,000 × *g* at 4°C for 2 hours using a Beckman SW32 or SW28 rotor, respectively (Beckman Coulter, Brea, CA, USA). Supernatants were discarded, and the pellets were resuspended in PBS (pH = 7.4) and stored at −80°C until further use. Vaccine titers were measured by 50% of egg infectious dose (EID_50_), plaque assay, and RT-qPCR.

### Hemagglutination assay

Turkey red blood cells (Lampire Biological Laboratories, Pipersville, PA, USA) were diluted with PBS to a final concentration of 0.5% (vol/vol) in agreement with previous work (3). In a 96-well V-bottom plate, 100 µL of AF was added to the first well of each row, and 50 µL of PBS was added to the rest of the wells, then serial twofold dilutions were performed by transferring 50 µL in each dilution step. After discarding the excess volume of the final dilution, 50 µL of the red blood cell suspension (0.5%) was added to all wells, and plates were read after 45 minutes of incubation at 4°C (15, 16). In-house produced NDV-HXP-S vaccines were used as positive controls.

### NP gene one-step reverse transcriptase-quantitative polymerase chain reaction

NDV primers targeting the NP gene were designed for the RT-qPCR. Primers were designed using the primer-BLAST online tool from NCBI. Forward primer: 5′-AGAGAGCACAGAGATTTGCG-3′ and reverse primer: 5′-GATCCTCTCCAGGGTATCGGT -3′ were used to amplify an amplicon of 128 base pairs (bp). RT-qPCR was performed using the iTaq Universal SYBR Green One-Step Kit (Bio-Rad Laboratories, Inc, Hercules, CA, USA) using the CFX Opus 96 Real-Time PCR Instrument (Bio-Rad Laboratories, Inc, Hercules, CA, USA). The master mix for a single PCR reaction consisted of 10 µL of the iTaq Universal SYBR Green reaction mix, 3.75 µL of nuclease-free water, 0.5 µL of each primer at a concentration of 10 µM, 0.25 µL of the iScript Reverse Transcriptase, and 5 µL of the template RNA. The RT-qPCR was run as follows: 50°C for 10 minutes, 95°C for 1 minute, and 40 consecutive cycles of 95°C for 10 seconds and 55°C for 20 seconds. No template control (NTC) with 5 µL of nuclease-free water as RNA sample was used as negative control. For each RT-qPCR run, a positive control consisting of a defined concentration of the template, derived from the plasmid used to generate the standard curve, was used. From the standard curve, the copies in 5 µL were interpolated, then the copies/μL were calculated and corrected by the amount of RNA sample extracted using the QIAmp Viral RNA Mini Kit. A plasmid containing the sequence of the NP was used as DNA standard (Twist Bioscience, South San Francisco, CA, USA). An 8-point, 10-fold dilution series of standard DNA was used to prepare the standard curve (11). The copies/μL were calculated as follows:


(1) $$\text{DNA}\left(\frac{\text{copies}}{\mu L}\right)=\frac{\text{DNA}\left(\frac{ng}{\mu L}\right)\times 10^{-9}\frac{g}{ng}\times 6.022\times 10^{23}\frac{\text{molecules}}{\text{mole}}}{N\left(bp\right)\times 650\frac{g}{\text{mole of bp}}}$$

### NP gene reverse transcriptase-polymerase chain reaction

Viral RNA was extracted using the QIAmp Viral RNA Mini Kit (Qiagen, Hilden, Germany) following the manufacturer’s instructions. A reverse transcriptase-polymerase chain reaction (RT-PCR) was performed using the SuperScript IV One-Step RT-PCR System (Thermo Fisher Scientific, Waltham, MA, USA) to amplify the transgene of the NP gene. Forward primer: 5′ - AGAGAGCACAGAGATTTGCG - 3′ and reverse primer: 5′ - GAGAGGCTACTAAGTGCAAGG -3′ were used to amplify an amplicon of 495 bp. Primers were used at a concentration of 10 µM. The RT-PCR was run as follows: 60°C for 10 minutes; 98°C for 2 minutes; 40 consecutive cycles of 98°C for 10 seconds, 63°C for 10 seconds, and 72°C for 20 seconds; and 72°C for 5 minutes. An agarose gel at 1% (wt/vol) with SYBR Safe (Invitrogen, Waltham, MA, USA) was performed to visualize the amplicon. The RT-PCR product was mixed with gel loading dye Orange DNA Loading Dye (1×, final concentration) and loaded to the gel. Ten microliters of Trackit 100 bp DNA Ladder (Thermo Fisher Scientific, Waltham, MA, USA) was added to one well to confirm the size of the resultant amplicon. Gel electrophoresis results were visualized using the Bio-Rad ChemiDoc Imaging System (Bio-Rad Laboratories, Inc, Hercules, CA, USA).

### NP gene RT-qPCR assay validation

RT-qPCR was validated following the ICH guideline M10 on bioanalytical method validation and study sample analysis (12) and the recommendations by the FDA guidance for gene therapy clinical trials and long-term follow-up after administration of human gene therapy products (13). The RT-qPCR assay was validated following established acceptance criteria described in Table 1, including specificity (no amplification in no template controls), linearity (*R*² >0.98), efficiency between 90% and 110%, sensitivity with a lower limit of detection ≤10 copies, and quantification ≤50 copies. Reproducibility was confirmed with intra-assay variation and inter-assay variation. Validation included five RT-qPCR runs performed over a minimum of 3 days by two different operators. Each plate contained one set of standards and NTC as negative control, all of them run in triplicates. The standard curve was used to evaluate the identity, linearity, sensitivity, specificity, and reproducibility of the qPCR assay.

**TABLE 1:** Acceptance criteria for qPCR-based assay validation (12, 13)

Acceptance criteria for RT-qPCR validation	
Specificity, linearity, and sensitivity	No template control wells should be blank
	Efficiency should be between 90% and 110%
	*R*^2^ >0.98
	Lower limit of detection ≤10 copies Lower limit of quantification ≤50 copies
Reproducibility (intra-assay precision)	Coefficient of variation of the Cq value between each standard replicate should be <2%
Reproducibility (inter-assay precision)	Coefficient of variation of the log (copies) of each standard from different validation runs should be <25%

#### Specificity

Specificity was determined by the lack of amplification in NTC in all the validation runs as well as evaluated *in silico* using SnapGene software (GSL Biotech LLC, Boston, MA, USA) by aligning the primer sequences against different NDV variants and virus of the Paramyxoviridae family. NP gene sequences were retrieved from the NCBI nucleotide database. NP sequences of NDV vaccine strains are described in Table 2, and NP sequence for the different paramyxoviruses is shown in Table S2. NP gene identity scores (%) between each pair of viruses were quantified using the two-sequence alignment tool of SnapGene software (GSL Biotech LLC, Boston, MA, USA).

**TABLE 2:** NCBI accession number of the NP gene sequences of different lentogenic, mesogenic, and apathogenic enteric NDV vaccine strains^*a*^

Virulency	NDV vaccine strains	NCBI accession number (nucleotide positions of NP gene CDS)
Lentogenic	La Sota	KC844235.1 (122 …1591)
	VG/GA	EU289028.1 (122 … 1591)
	F	KC987036.1 (122 … 1591)
	Hitchner B1	AF060483.1 (7 … 1476)
Mesogenic	Mukteswar	EF201805.1 (122 … 1591)
	Komarov	EF442115.1 (1 … 1470)
	Roakin	AF419409.1 (67 … 1536)
Apathogenic enteric	VH	KM056358.1 (122 … 1591)
	Ulster 2C	Z30084.1 (67 … 1536)
	V4	JX524203.1 (122 … 1591)

^*a*^ The nucleotide positions shown correspond to the coding sequence of the nucleoprotein gene within each genome.

#### Linearity

Linearity was determined using a suitable linear regression analysis of the Cq value vs NP gene log copies. Slope, efficiency, and the coefficient of determination ($R^{2}$) were calculated using Maestro Software (Bio-Rad).

#### Sensitivity

The sensitivity was determined by the lowest limit of quantification (LLOQ) and lowest limit of detection (LLOD). The LLOQ and LLOD were established according to the FDA recommendations as 50 copies/target and 10 copies/target, respectively (13). The upper limit of detection was established by the highest point in the standard curve. To calculate the empirical LLOD, serial dilutions of NDV-HXP-S were performed in technical triplicates and quantified by RT-PCR, RT-qPCR, and HA assay.

#### Reproducibility

The intra-assay precision was determined by analyzing the coefficient of variation (CV) of the Cq values of the intra-assay replicates, with an acceptance threshold of <2%, according to the acceptance criteria. The inter-assay precision was determined by analyzing the CV of the log copies between the inter-assay replicates in five independent runs, with an acceptance threshold of <25%, also in-line with these guidelines.

### Fifty percent of egg infectious dose (EID_50_)

EID_50_ quantification was performed in 8–10 days old embryonated chicken eggs. NDV-HXP-S samples were 10-fold serially diluted in PBS, resulting in 10^−7^–10^−12^ dilutions of the virus. One hundred microliters of each dilution was injected into each egg for a total of six eggs per dilution. The eggs were incubated at 37°C according to each virus growth condition and then cooled at 4°C overnight. AF was collected and analyzed by HA assay. The EID_50_ titer of the NDV, determined by the number of HA-positive and HA-negative eggs in each dilution, was calculated using the Reed and Muench method (3).

### Next-Generation Sequencing

Next-Generation Sequencing (NGS) was performed using the Amplicon-EZ service from a commercial laboratory (Genewiz from Azenta Life Sciences, South Plainfield, NJ, USA). Primers targeting a specific region of the RBD spike protein with variability between all the variants were designed including NGS-adapter sequences (forward primer: 5′-ACACTCTTTCCCTACACGACGCTCTTCCGATCTCTACCAAGCTGAACGACCTGTGCTTCAC-3′, reverse primer: 5′-GACTGGAGTTCAGACGTGTGCTCTTCCGATCTAGTTCGAAGCTCAGCACCACCACTCTGTA

-3′. A variable region within the receptor-binding domain of the spike protein, which allows differentiation among all variants of concern, was first identified. Conserved flanking sequences surrounding this region were then selected for primer design. Primer specificity was confirmed *in silico* by aligning the primer sequences using SnapGene software (GSL Biotech LLC, Boston, MA, USA). An RT-PCR was performed using the SuperScript IV One-Step RT-PCR System (Thermo Fisher Scientific, Waltham, MA, USA). Forward and reverse primers were used for the RT-PCR at a concentration of 10 µM. RT-PCR was performed as follows: 50°C for 10 minutes; 98°C for 2 minutes; 40 consecutive cycles of 98°C for 10 seconds, 72°C for 10 seconds, and 72°C for 20 seconds; and 72°C for 5 minutes. RT-PCR products were purified using the PCR Clean-up protocol of the NucleoSpin Gel and PCR Clean-up (Macherey-Nagel, Düren, Germany). Samples were then normalized to 20 ng/µL and sent to a commercial laboratory for NGS (14). Full sequence coverage was obtained by paired-end data with two sequencing fastq files per sample. Files were extracted from the compressed folder and analyzed using the SeqMan Ultra software (DNA STAR, Madison, WI, USA), using the variant analysis workflow for NGS amplicon data. Settings were modified to obtain the highest specificity. Read technology was Illumina paired-end data, with multisample experiment setup. Mer size was changed to 31 nucleotides (nt), and the minimum match percentage was changed to 100%. Minimum aligned length was also modified to 200 nt. In the analysis options, detection of single nucleotide polymophisms (SNPs) and other small variants was unchecked, with a 100% match threshold; no mismatches or variants are expected to be present in the aligned reads. The number of sequences of each variant assembly over the total number of sequences was measured to estimate the frequency of each variant in the sample.

### NGS assay validation

NDV-HXP-S variant vaccines expressing the spike of Ancestral, Beta, Gamma, Delta, BA.1, BA.5, BQ.1.1, and XBB.1.5 were used to validate the technique. RNA was extracted using the QIAmp Viral RNA Mini Kit (QIAGEN, Hilden, Germany) following the manufacturer’s instructions. RT-PCR was performed as explained above, in the NGS section of Materials and Methods. The specific criteria used in this study are described in Table 3. Each variant vaccine sample was run by technical duplicate.

**TABLE 3:** Acceptance criteria for NGS-based assays validation

Acceptance criteria for NGS validation	
Specificity, linearity, and sensitivity	Detection only of codon-optimized NDV-HXP-S RBD amplicons
	Empirical error of 10% is accepted
	*R*^2^ >0.98
	Distinguish between sequences with just 1 nt difference
Reproducibility (intra-assay and inter-assay precision)	Coefficient of variation of the relative percentage of sequences of each amplicon from samples of different runs should be <25%

#### Specificity and sensitivity

The specificity of the technique was evaluated by performing *in silico* and *in vitro* analyses. Primers were aligned against the S protein of SARS-CoV-2 (NCBI Gene ID: 43740568) and the codon-optimized S protein in the different NDV-HXP-S variant vaccines *in silico* using SnapGene software (GSL Biotech LLC, Boston, MA, USA). *In vitro* amplification of individual amplicons for Ancestral, Beta, Gamma, Delta, BA.1, BA.5, BQ.1.1, and XBB.1.5 vaccines was performed, and the relative percentage of each variant was analyzed by NGS. For each variant, at least one distinguishing nucleotide difference and a relative frequency above 90% in the sequencing data were expected. The relative error was measured using equation 2, calculated by comparing the measured frequency to the expected frequency for each variant in the sample. NGS analysis was performed on samples with known amounts of NDV-HXP-S variant amplicons. Specifically, individually sequenced variant vaccines and mixed samples containing defined proportions of Ancestral and XBB.1.5 amplicons were used for the analysis.


(2) $$Relative\;error=\;\frac{Expected\;value-Real\;value}{Real\;value}\;x\;100$$

#### Linearity

Linearity was studied by analyzing samples containing different proportions of two amplicons (Ancestral and XBB.1.5). Linear correlation between the predicted and the observed values was analyzed and compared to the acceptance criteria.

#### Reproducibility

The intra-assay precision was determined by analyzing the CV of the relative frequency of each variant vaccine measured in duplicate. The inter-assay precision was determined by analyzing the CV of Ancestral amplicon measured at three different times.

### *In vitro* infections

Vero-E6 (CRL-1586) cells were used to evaluate *in vitro* replication of NDV-HXP-S monovalent and multivalent formulations. Briefly, Vero-E6 cells were seeded onto 12-well plates in growth media (1% HEPES 1M, 1% penicillin-streptomycin, and 10% heat-inactivated fetal bovine serum in Dulbecco’s modified Eagle medium [DMEM]) and were cultured for 1 day. Once the cells achieved 80%–100% confluency, they were infected with monovalent Ancestral, Beta, Delta NDV-HXP-S vaccines or with the trivalent formulation (Ancestral + Beta + Delta) described in previous work (17), at an MOI of 0.1 in biological triplicates. Infection was carried out in infection media containing 1% HEPES 1M, 1% penicillin-streptomycin, and 0.3% Bovine Serum Albumin (BSA) in DMEM, and N-tosyl-L-phenylalanine chloromethyl ketone (TPCK)-trypsin at 1 µg/mL to allow for multicycle infection. Seventy-two hours post infection (hpi), lysates and supernatants were collected. Viral RNA was extracted using the QIAmp Viral RNA Mini Kit (QIAGEN, Hilden, Germany) for the supernatant following the manufacturer’s instructions and the PureLink RNA Mini Kit (Thermo Fisher Scientific, Waltham, MA, USA) for the lysates.

## RESULTS

### NDV nucleoprotein sequence identity among NDV strains

**Fig 2:**
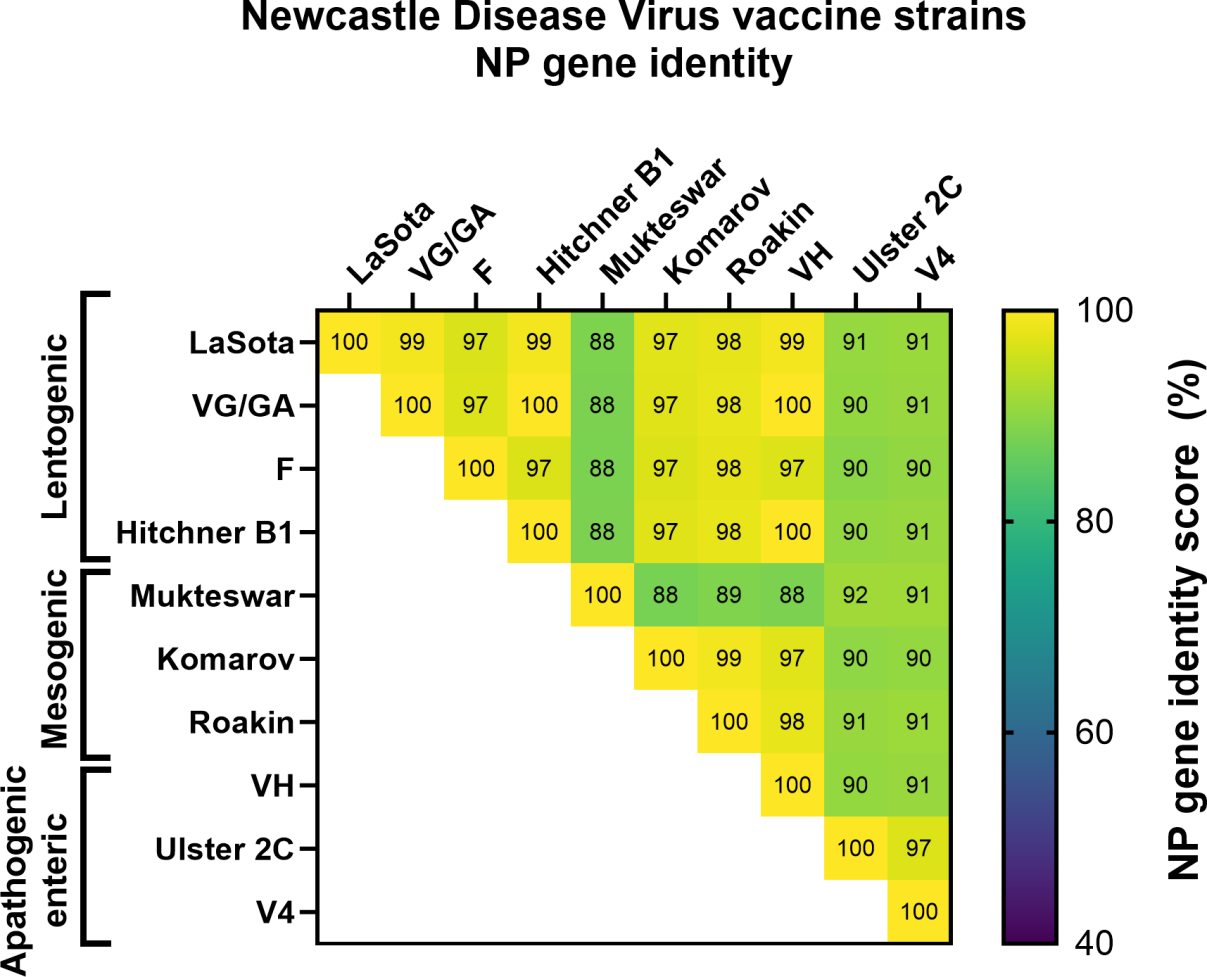
Nucleoprotein sequence identity among NDV vaccine strains. Reference sequence of lentogenic (VG/GA, F, and Hitchner B1), mesogenic (Mukteswar or Komariv), and apathogenic enteric NDV strains (VH, Ulster 2C, and V4) was extracted from the NCBI website, and two DNA sequence alignments were performed using the Smith-Waterman method to calculate the identity score.

The NP gene was chosen as the target to monitor NDV presence in biological samples. NP is the most abundant component of the nucleocapsid complex (composed of NP, L, and P) and has two conserved functions among paramyxoviruses: protect the viral genomic RNA and assist in the viral RNA synthesis (18). To ensure the specificity of our RT-qPCR method, we studied the NP sequence identity *in silico* among representative viruses of the Paramyxoviridae family (Fig. S1). Lower identity scores ranging from 46% to 58% were found for the NP gene within the Paramyxoviridae family, supporting only the detection of NDV-like viruses when the NP gene is targeted. We then performed the same *in silico* analysis among different NDV strains, including several lentogenic (VG/GA, F, and Hitchner B1), mesogenic (Mukteswar or Komarov), and apathogenic enteric strains (VH, Ulster 2C, and V4) (19, 20). An NP sequence identity higher than 90% was observed for all strains evaluated, except for the Mukteswar strain (Fig. 2). Next, we designed a set of primers targeting a conserved region in the NP gene of 128 bp. Further i*n silico* analyses with the NDV NP reference strains (Table S3) and other NP paramyxoviruses (Table S4) confirmed only NP primer annealing with NDV sequences and not with the other viruses of the family.

### Validation of the NDV NP gene RT-qPCR method

Using a recombinant plasmid DNA encoding for LaSota NDV NP sequence, we optimized an RT-qPCR assay to meet the acceptance criteria defined in Table 1. We chose to use SYBR Green since it is a quick, cheap, and reliable method that can be easily implemented in several clinical settings (21). Results from five independent runs were used to evaluate the linear range, reproducibility, and intra- and inter-assay precision of the method. A dynamic range of 10^8^–10^1^ copies was used for these assays. The results from the five runs are depicted in Table 4, where all runs met the linearity acceptance criteria, and no amplification was shown in the NTC.

**TABLE 4:** RT-qPCR linearity^*a*^

Validation runs	Efficiency (%)	*R* ^2^	Slope	*y*-int	No template control
1	100.10	0.99	−3.318	38.404	Blank
2	98	0.987	−3.371	39.421	Blank
3	96.3	0.996	−3.414	40.749	Blank
4	103.4	0.993	−3.243	38.46	Blank
5	108	0.989	−3.144	38.254	Blank

^*a*^ Efficiency,* R*^2^ , slope, and *Y*-intercept (*y*-int) of five independent runs. All runs meet the requirements of the acceptance criteria.

Intra-assay precision was evaluated by quantifying the coefficient of variation of technical triplicates. A CV below 2% was obtained in all samples analyzed in agreement with intra-precision acceptance criteria (Table 5). Inter-assay precision and reproducibility were calculated by analyzing the CV of the NDV genome copies calculated after five independent runs performed in different days. The CV of log copies was below 25% in all standard samples analyzed, supporting the reproducibility of the method (Table 6).

**TABLE 5:** RT-qPCR intra-assay precision^*a*^

	RUN 1	RUN 2	RUN 3	RUN 4	RUN 5
Standard-1	0.50%	0.28%	0.63%	0.65%	0.23%
Standard-2	0.41%	0.44%	0.39%	0.13%	0.25%
Standard-3	0.32%	0.95%	0.54%	0.92%	0.06%
Standard-4	0.79%	0.26%	0.01%	0.19%	0.17%
Standard-5	0.68%	0.61%	0.29%	0.43%	0.28%
Standard-6	0.26%	0.34%	0.67%	0.61%	0.86%
Standard-7	1.07%	0.66%	1.28%	0.80%	0.05%

^*a*^ Coefficient of variation of the Cq values at each dilution (standards 1–7) in five independent runs. All standard dilutions were run in triplicates. In all runs, the CV was less than 2%, meeting the acceptance criteria.

**TABLE 6:** RT-qPCR inter-assay precision^*a*^

	Standard-1	Standard-2	Standard-3	Standard-4	Standard-5	Standard-6	Standard-7
Mean	7.83	6.91	6.09	5.21	4.15	3.04	1.91
SD	0.14	0.06	0.22	0.09	0.13	0.07	0.13
CV	1.76%	0.89%	3.68%	1.78%	3.18%	2.42%	6.87%

^*a*^ Mean, SD, and coefficient of variation of the log copies at each dilution among the five independent runs. CV of the log copies is less than 25%, meeting the acceptance criteria.

### Determination of RT-qPCR empirical lower limit of detection

**Fig 3:**
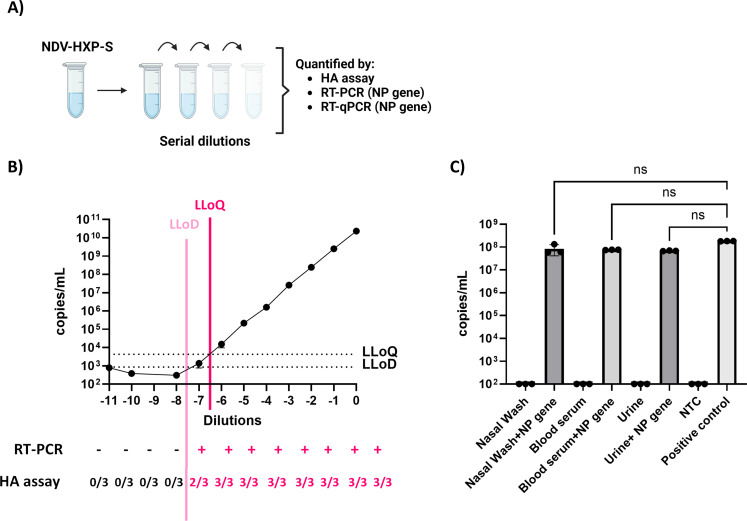
Determination of RT-qPCR empirical lower limit of detection and matrix effect. (**A**) The lower limit of detection was established through serial dilutions of LaSota NDV. Each dilution was quantified by HA assay, EID_50_, RT-PCR, and RT-qPCR. LLOD was set by the dilution that showed lack of detection of NDV. Figure created with Biorender. (**B**) In all techniques, the dilution which showed lack of detection of NDV was dilution −8, which corresponds to a Cq value of 36. LLOQ is established as 50 copies. (**C**) Specificity was validated by the lack of matrix effect in the biological tissues. The matrix effect was tested by comparing negative biological fluids spiked and non-spiked with a known concentration of target RNA. No non-specific amplification or amplification inhibition was detected. Kruskal-Wallis analysis between conditions is shown (*P* > 0.05 ns).

Internal LLOD and LLOQ were established as 10 copies and 50 copies per reaction, respectively, following FDA recommendations (13). Empirical LLOD was determined by comparing the minimum detectable NP copies compared to gold-standard NDV analytical methods: PCR, HA assay after infection in SPF eggs, and EID_50_ quantification (Fig. 3A). An NDV-HXP-S vaccine preparation was used for these analyses. Eleven 10-fold dilutions were performed in PBS, and the different virus dilutions were quantified with all four methods. In all cases, the LLOD was encountered at the same dilution as shown in Fig. 3B: dilution −7 presented the minimum amount of 10 copies per reaction that translated into 8.57 × 10^10^ copies/mL. DNA electrophoresis after RT-PCR presented a positive band until the same dilution, and two out of three eggs were HA positive after SPF infection. EID_50_ titration calculated from the HA assay gave a titer of 1.78 × 10^8^ EID_50_/mL.

Next, we studied the matrix effect of different human biological fluids. Viral RNA was extracted from different biological fluids, including saliva, nasal swabs, and urine samples, and a known concentration of NDV NP gene was spiked into those samples. Neither non-specific amplifications nor inhibition effects were observed in any of the conditions tested (Fig. 3C), confirming the robustness of the method in detecting the presence of the NDV in different biological fluids.

### Development and validation of an RBD-based NGS method to quantify NDV-HXP-S variant vaccines

**Fig 4:**
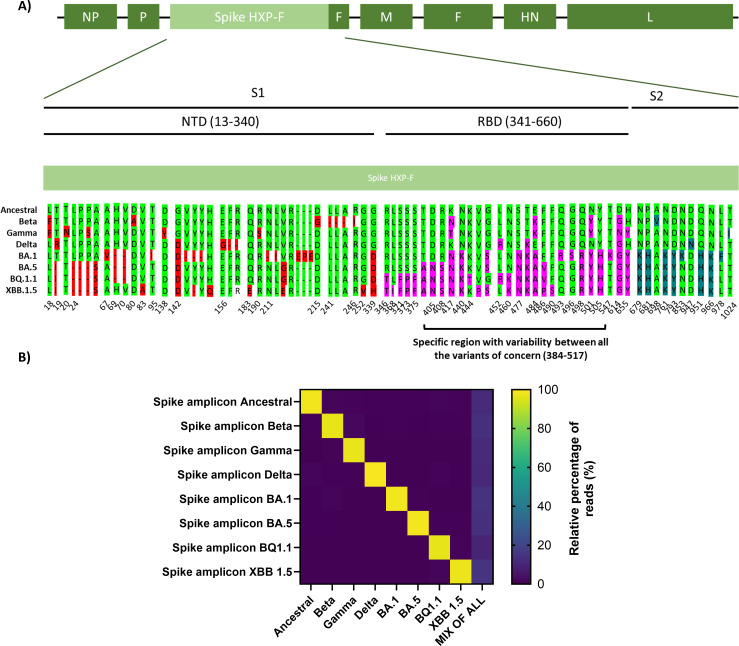
Development and validation of an RBD-based NGS method to quantify NDV-HXP-S variant vaccines. (**A**) NDV-HXP-S genome. Amino acid spike sequence of each variant of concern using the NDV-HXP-S Ancestral numbering. Mutations compared to the Ancestral spike genome are depicted for each VOC. Mutations located in the N-terminal domain (NTD), the receptor-binding domain, and the S2 subunit are shown in red, pink, and blue, respectively. The highly variable region (384–517) used in the NGS method is depicted. (**B**) Heatmap representing the relative percentage of reads of each variant amplicon present in the sample. The *Y*-axis represents the reference amplicon sequences and the *X*-axis the analyzed sample. On average, 47,771 reads were aligned per sample.

**Fig 5:**
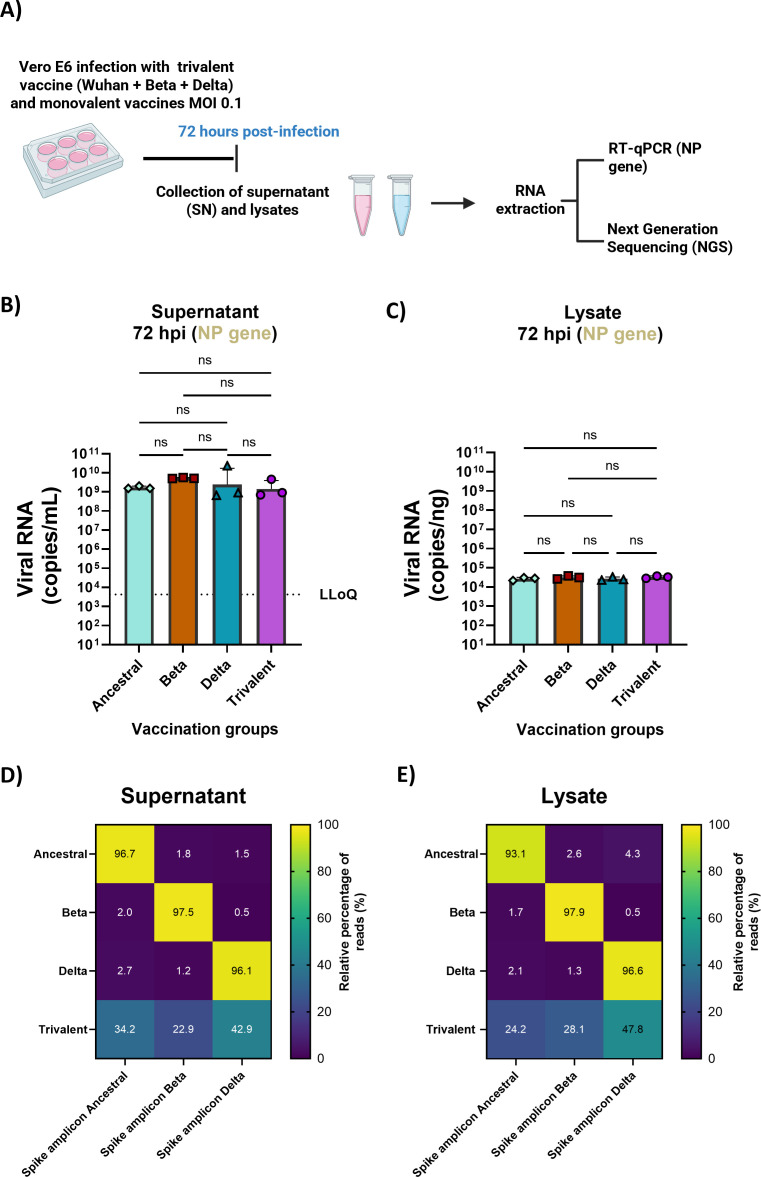
Application of NDV NP RT-qPCR method and RBD-based NGS in *in vitro* cell cultures. (**A**) Experimental outline. Vero E6 cells were infected with NDV-HXP-S monovalent vaccines (Ancestral, Beta, and Delta) and a trivalent vaccine composed of the three variants. Media were supplemented with 1  µg/mL TPCK trypsin to allow for multicycle infection. Seventy-two hours post infection, supernatant (SN) and lysates were collected, and RNA was extracted to perform NP gene RT-qPCR and sent to next-generation sequencing for variant analysis. Figure created with Biorender. (**B, C**) NP gene RNA copies/mL for SN and copies/ng for lysate samples. Results show similar amounts of NP gene copies in the trivalent and monovalent infections. Kruskal-Wallis analysis between conditions is shown (*P* > 0.05 ns). (**D, E**) Heatmap representing the relative percentage of each variant present in the sample. The *Y*-axis represents the samples and the *X*-axis the reference amplicon sequences. Each sample was run in triplicate. The mean of the three replicates is shown in the heatmap. On average, 48,761 reads were aligned per sample.

An NGS method was generated to differentiate between NDV-HXP-S variant vaccines. A highly variable region in the RBD of the S protein was chosen for this end as shown in Fig. 4A. Amplicon-based NGS was performed using Illumina sequencing in a bidirectional way (14). The amplicon-based NGS generated ~50,000 reads in depth sequencing with an average length of 250 bp. Afterward, the reads were aligned against the different reference genomes to calculate the relative percentage of sequences that match with each template. To do so, DNAStar software SeqMan Ultra (DNA STAR, Madison, WI, USA) using the variants analysis method was used. To validate the RBD-amplicon-based NGS, purified NDV-HXP-S variant vaccines were quantified by this method in duplicate. In all cases, NDV-HXP-S variant vaccines presented a frequency greater than 90% against its homologous sequence, confirming the precision and sensitivity of the method. The CV of the technical duplicates was 1%, indicating minimal variability between replicates and confirming the high intra-assay reproducibility of the technique. To determine the empirical relative error, sequencing was performed on samples with known variant proportions: individual NDV-HXP-S variants (100% of a single variant) and mixtures of NDV-HXP-S Ancestral and XBB.1.5 amplicon sequences at defined ratios (Table 7). The relative error for each sample was calculated by comparing the theoretical and empirical variant frequencies, and the average error across all samples was 5% ± 4%. To further evaluate the sensitivity of the technique, purified NDV-HXP-S amplicons from Ancestral, Beta, Gamma, Delta, BA.1, BA.5, BQ.1.1, and XBB.1.5 were mixed in equal amounts, with a final theoretical concentration of 12.5% per variant (Fig. 5B, last column). A percentage of 11.5%, 13.6%, 11.4%, 11.1%, 14.4%, 13.5%, 9.7%, and 14.8% was obtained for each variant, respectively, confirming the differentiation of each variant from the complex pool.

**TABLE 7:** Theoretical vs observed number of sequences (%) obtained for the theoretical mix of Ancestral and XBB.1.5 NDV-HXP-S amplicons

Ancestral NDV-HXP-S amplicon (%)		XBB 1.5 NDV-HXP-S amplicon (%)	
Theoretical	Observed	Theoretical	Observed
50	46	50	53
75	66	25	34
87.5	79	12.5	21
93.65	86	6.25	14
96.8	91	3.2	9

NDV-HXP-S Ancestral and XBB.1.5 amplicon sequences were used to evaluate the linearity of the technique. Samples mixing different proportions of each amplicon were analyzed (Table 7), and a linear correlation analysis between the theoretical and the empirical values was performed. A significant linear correlation with an $R^{2}$ of 0.99 was achieved, supporting the linearity of the method. Finally, the robustness (inter-assay of the method) was evaluated by performing three independent runs of the Ancestral amplicon sequences. A CV of 2% was achieved (Table 8), meeting the acceptance robustness criteria as shown in Table 3.

**TABLE 8:** Inter-assay precision^*a*^

Ancestral NDV-HXP-S amplicon replicate	Relative percentage of reads (%)	Mean (%)	SD (%)	CV (%)
1	99.52	98	0.015	2
2	99.2			
3	96.72			

^*a*^ Mean, SD, and coefficient of variation of the relative percentage of sequences of same amplicon analyzed in three different days. CV is less than 25%, meeting the requirements of the acceptance criteria. On average, 43,392 reads were aligned per sample.

### Application of NDV NP RT-qPCR method and RBD-based NGS in *in vitro* cell cultures

To evaluate the application of both techniques, Vero E6 cells were infected with monovalent Ancestral, Beta, Delta NDV-HXP-S vaccines or with the trivalent formulation described in previous work (17), at an MOI of 0.1 in biological triplicates (Fig. 5A). Seventy-two hours post infection, cell lysates and supernatants were collected and analyzed by RT-qPCR to measure the NP viral genome copies and by NGS to study the relative percentage of each variant vaccine when administered together.

Comparable NP viral copies/mL were obtained after the monovalent and the trivalent infections 72 hpi (Fig. 5B). Regarding the variant analysis, monovalent vaccines contained its homologous sequence in relative frequencies higher than 90% (Fig. 5C). This relative percentage was similar to the one obtained in the NGS validation experiments, supporting the use of this technique in *in vitro* models. As for the trivalent infection, a relative frequency of 33% is expected to conclude that the three variants replicate equally when administered together. Results show the relative percentage of reads of each variant was 34.2%, 22.9%, and 42.9% at an MOI of 0.1 in cell supernatants for Ancestral (Wuhan), Beta, and Delta variants (Fig. 5C), supporting the replication of the three variants when co-administered together.

## DISCUSSION

Since the declaration of the COVID-19 pandemic, huge efforts have been undertaken to develop effective vaccines as well as reliable diagnostic methods. In previous work, our groups have developed NDV-based viral vector vaccines (NDV-HXP-S) to prevent SARS-CoV-2 infections (3). NDV is attenuated in mammalian hosts (4, 22, 23), making it a promising live-attenuated vector that can be administered as mucosal vaccine. With views to study the viral shedding of these new candidates in monovalent and multivalent formulations, we developed two methods to quantitatively assess NDV-based vaccines *in vitro* and *in vivo*.

Nucleic acid amplification tests are globally accepted as one of the gold-standard detection methods for respiratory viruses, including SARS-CoV-2 and influenza viruses (24). Like influenza, NDV is a negative-stranded RNA virus; hence, it requires the synthesis of positive-stranded mRNA sequences to produce the different viral proteins (25). With the aim to detect any possible presence of the virus, we designed an RT-qPCR assay, in which the reverse transcriptase reaction will amplify both positive and negative strands. We chose to target a small region of the NP protein, which is a conserved and abundant structural protein among NDV vaccine strains, like LaSota or B1 (26, 27). An RT-qPCR efficiency of 100% ± 10% was obtained in the validation with an *R*^2^ >0.98, which confirmed the linearity of the method. The inter- and intra-assay CV values were below 25% and 2%, respectively, supporting the good precision and reproducibility of the technique. NDV isolation has been classically performed by infection of SPF-embryonated chicken eggs (28). Although the method is known to be highly sensitive, it is labor intensive. The suspected material, which typically comes from avian respiratory secretions or cloacal samples, needs to be mixed with an antibiotic cocktail and incubated for several days in SPF eggs (28). Comparing the technology involving the infections of SPF-embryonated chicken eggs and the current RT-qPCR technique revealed the same value for LLOD. We believe the current RT-qPCR methodology presents great promise to quantify NDV viral copies in a short period of time without the need for antibiotic cocktails or egg supplies.

With the aim of testing a mucosal multivalent NDV-HXP-S vaccine, we developed an NGS method to quantify the relative percentage of different NDV-HXP-S variant vaccines in a single formulation. We designed primers flanking a specific region of the spike protein with high variability between all the current VOCs (Ancestral, Beta, Gamma, Delta, BA.1, BA.5, BQ.1.1, and XBB.1.5). Of note, we have also tested the method with the newly developed EG.5, JN.1, and KP.2 NDV-HXP-S vaccines (data not shown). Importantly, the targeted region includes variant-defining nucleotide differences, and the method can distinguish variants differing by as little as a single nucleotide, which supports its high accuracy even in co-infection scenarios. The assay showed high specificity allowing to differentiate each variant from the rest with an average error of 5% ± 4%. In *in vitro* cell culture experiments, monovalent NDV-HXP-S infections were detected with a specificity of 93%–98%, whereas trivalent NDV-HXP-S formulations presented similar amounts (22.9%–42.9%) of each spike in the cell supernatants analyzed 72 hours post infection. In conclusion, both techniques were validated to accurately quantify NDV-HXP-S vaccines in monovalent and multivalent formulations. Of note, other NDV-based vaccines against respiratory infections, including influenza A (29), human parainfluenza virus-3 (30) or respiratory syncytial virus (31), among others, are in preclinical development (19). The present results will help the study of virus shedding in future clinical trials to evaluate mucosal NDV-based vaccines.

## Data Availability

Raw data can be found in ImmPort under the identifiers: SDY3294

## SUPPLEMENTAL MATERIAL

Figures S1 and Table S1 to S4 spectrum.00653-25-s0001.pdf Figure S1: NP sequence identity in silico among representative viruses of the paramyxoviridae family. Table S1: Specific growth conditions for each NDV-HXP-S viral vaccine. Table S2: NCBI accession number of the NP gene sequences of different representative viruses of the paramyxoviridae family. Table S3: NP primers alignment with NDV vaccine strains. Table S4: NP primers alignment with paramyxoviridae family.

ASM does not own the copyrights to Supplemental Material that may be linked to, or accessed through, an article. The authors have granted ASM a non-exclusive, world-wide license to publish the Supplemental Material files. Please contact the corresponding author directly for reuse.
