# Supplementary material for: Development of an RT-qPCR and a Next-Generation Sequencing approach to assess viral shedding of NDV-based SARS-CoV-2 variant vaccines
Source: Microbiol Spectr. 2025 Nov 25;14(1):e00653-25. doi: 10.1128/spectrum.00653-25 (PMC12772256; doi:10.1128/spectrum.00653-25)
Supplement: Figures S1 and Table S1 to S4 — Figure S1: NP sequence identity in silico among representative viruses of the paramyxoviridae family. Table S1: Specific growth conditions for each NDV-HXP-S viral vaccine. Table S2: NCBI accession number of the NP gene sequences of different representative viruses of the paramyxoviridae family. Table S3: NP primers alignment with NDV vaccine strains. Table S4: NP primers alignment with paramyxoviridae family. [file spectrum.00653-25-s0001.pdf]

Supplementary material

Development of an RT-qPCR and a Next Generation Sequencing approach to assess viral shedding of NDV-based SARS-CoV-2 variant vaccines

Marta Boza[1], Adam Abdeljawad[1], Stefan Slamanig[1,2], Nicholas Lemus[1], Tsoi Ying Lai[1], William Shea[1], Weina Sun[1], Peter Palese[1,3*], Irene González-Domínguez[1*]

[1] Department of Microbiology, Icahn School of Medicine at Mount Sinai, New York, NY 10029, USA

[2]Swammerdam Institute for Life Sciences, University of Amsterdam, Amsterdam, The Netherlands

[3] Department of Medicine, Icahn School of Medicine at Mount Sinai, New York, NY 10029, USA

Supplementary Table 1: Specific growth conditions for each NDV-HXP-S viral vaccine.

NDV-HXP-S VIRAL STOCKS	DILUTION (log10)	INCUBATION TIME (hours)
**Wuhan**	-6	96
**Beta**	-6	96
**Gamma**	-6	72
**Delta**	-6	120
**BA.1**	-6.5	120
**BA.5**	-5.5	96
**BQ.1.1**	-6	120
**XBB.1.5**	-6	96

Supplementary Table 2: NCBI accession number of the NP gene sequences of different representative viruses of the paramyxoviridae family.

GENUS	VIRUS	NCBI WHOLE GENOME ACCESION NUMBER	GENE ID
Orthoavulavirus	*Avian avulavirus 1* (Newcastle disease virus)	NC_039223.1	37627207
Metaavulavirus	*Avian avulavirus 2* (avian paramyxovirus 2)	NC_039230.1	37627230
Paraavulavirus	*Avian avulavirus 3* (avian paramyxovirus 3)	NC_025373.1	20964506
Orthorubulavirus	*Mumps rubulavirus* (mumps virus)	NC_002200.1	1489766
Metarubulavirus	*Menangle rubulavirus* (Menangle virus)	NC_039197.1	37626952
Respirovirus	*Human respirovirus 1* (human parainfluenza virus 1)	NC_003461.1	935258
Aquaparamyxovirus	*Salmon aquaparamyxovirus* (Atlantic salmon paramyxovirus)	NC_025360.1	20964430
Ferlavirus	*Reptilian ferlavirus* (fer-de-lance virus)	NC_005084.2	2732797
Henipavirus	*Hendra henipavirus* (Hendra virus)	NC_001906.3	1446470
Jeilongvirus	*Beilong jeilongvirus* (Beilong virus)	NC_007803.1	3964367
Narmovirus	*Nariva narmovirus* (Nariva virus)	NC_017937.1	12977631
Morbillivirus	*Measles morbillivirus* (measles virus)	NC_001498.1	1489804
Salemvirus	*Salem salemvirus* (Salem virus)	NC_025386.1	21011778
Synodonvirus	*Synodus synodonvirus* (Wenling triplecross lizardfish paramyxovirus)	NC_075439.1	80527883

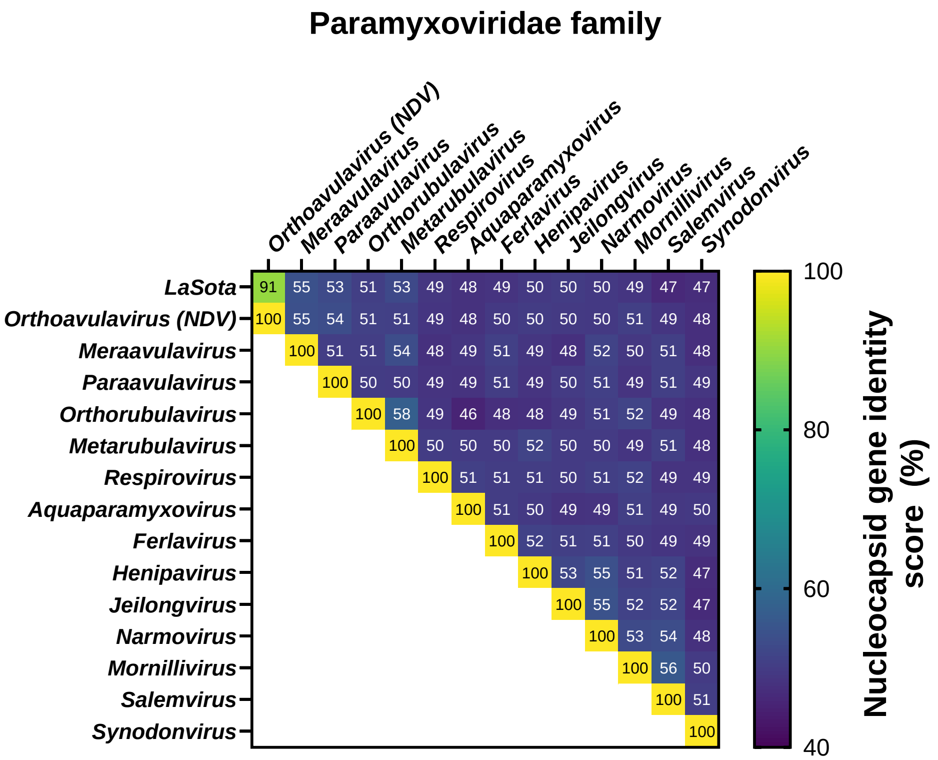

Supplementary Figure 1: NP sequence identity *in silico* among representative viruses of the paramyxoviridae family.

Supplementary Table 3: NP primers alignment with NDV vaccine strains.

NDV Vaccine strains	*FORWARD PRIMER*	*REVERSE PRIMER*
La Sota	Yes	Yes
VG/GA	Yes	Yes
F	1 mismatch	Yes
Hitchner B1	Yes	Yes
Mukteswar	4 mismatches	No
Komarov	1 mismatch	Yes
Roakin	1 mismatch	Yes
VH	Yes	Yes
Ulster 2C	3 mismatches	3 mismatches
V4	3 mismatches	3 mismatches

Supplementary Table 4: NP primers alignment with paramyxoviridae family.

PARAMYXOVIRIDAE FAMILY	*FORWARD PRIMER*	*REVERSE PRIMER*
Orthoavulavirus	No	No
Meraavulavirus	No	No
Paraavulavirus	No	No
Orthorubulavirus	No	No
Metarubulavirus	No	No
Respirovirus	No	No
Aquaparamyxovirus	No	No
Ferlavirus	No	No
Henipavirus	No	No
Jeilongvirus	No	No
Narmovirus	No	No
Mornivillivirus	No	No
Salemvirus	No	No
Synodonvirus	No	No
